# Improvement of Bladder Dysfunction by *Quisqualis indica* Extract in a Partial Bladder Outlet Obstruction Female Rat Model

**DOI:** 10.3390/ph19071040

**Published:** 2026-07-03

**Authors:** Jeongsook Kim, Jun-Yeop Song, Kyungmi Kim, Sang-Yoon Kim, Jae-Yong Kim, Poornima Kumbukgahadeniya, Hyo-Jung Kwun, Kyu Pil Lee

**Affiliations:** 1 Department of Veterinary Physiology, College of Veterinary Medicine, Chungnam National University, Daejeon 34134, Republic of Korea; 2 Department of Veterinary Pathology, College of Veterinary Medicine, Chungnam National University, Daejeon 34134, Republic of Koreaplakshinikg@o.cnu.ac.kr (P.K.);; 3 Huons Dongam R&D Center, Huons Co., Ltd., Gwacheon-si 13840, Republic of Korea

**Keywords:** *Quisqualis indica*, bladder dysfunction, partial bladder outlet obstruction (pBOO), urinary bladder, voiding

## Abstract

**Background:** Bladder dysfunction is a complicated condition that substantially impairs quality of life for both men and women. Due to the adverse effects and limited efficacy of current therapies, new strategies must be rapidly developed. Female bladder dysfunction arises from multifaceted etiologies distinct from the predominantly male benign prostatic hyperplasia (BPH) that is the focus of existing drug development. In this study, we investigated the therapeutic potential of *Quisqualis indica* extract (QIE), a traditional medicinal herb that attenuates BPH-induced lower urinary symptoms (LUTS), to elucidate its underlying mechanisms in a female bladder dysfunction model. **Methods and Results:** A bladder dysfunction model was established by inducing partial bladder outlet obstruction (pBOO) in female Sprague Dawley rats, followed by the oral administration of QIE for 7 weeks. Voiding pattern analysis and cystometry were conducted to evaluate indicators such as voiding frequency, voiding volume, and intravesical pressure. Histological analysis of excised bladder tissue quantified smooth muscle hypertrophy and collagen deposition. Gene expression profiling of inflammatory cytokines and fibrosis-related markers within the bladder tissue was performed to assess tissue remodeling. Furthermore, pharmacological contraction studies examined the direct effects of QIE on detrusor muscle responsiveness to muscarinic and purinergic agonists. QIE administration significantly improved the elevated voiding pressure and abnormal inter-contraction intervals observed in the pBOO rats, restoring normal voiding patterns. Histological examination revealed a marked decrease in muscle hypertrophy and collagen deposition. Expression levels of pro-inflammatory cytokines (TNFα, IL-1β) and fibrosis-associated genes (TGF-β, α-SMA) were downregulated. Pharmacological contraction assays demonstrated that QIE attenuated the hypercontractile response of bladder smooth muscle to a muscarinic agonist, with concurrent reduced expression of muscarinic receptors (M_2_, M_3_) at the mRNA level. **Conclusions:** *QIE* ameliorates key aspects of bladder dysfunction, voiding abnormalities, inflammation, fibrosis, and hypercontractility by modulating muscarinic receptor signaling and fibrotic pathways. This study suggests that QIE warrants further investigation as a natural product-based therapeutic candidate for female bladder dysfunction.

## 1. Introduction

Lower urinary tract (LUT) disorders, such as overactive bladder (OAB) and urinary incontinence (UI), are chronic conditions that significantly reduce quality of life across physical, psychological, social, and sexual domains [1]. These disorders are primarily characterized by symptoms such as frequent urination, nocturia, urgency, and dysuria. The underlying pathophysiological mechanisms are intricately intertwined, involving abnormal contractions of the bladder smooth muscle, urothelial inflammation and damage, and dysregulation of neural control [2,3,4].

Current therapeutic approaches to male bladder dysfunction target the well-defined anatomical basis of benign prostatic hyperplasia [5]. In contrast, treatment options for female patients with an overactive bladder predominantly rely on antimuscarinic agents or β3-adrenergic agonists that suppress excessive contractions of the bladder smooth muscle. However, these pharmacological interventions are frequently associated with adverse effects, including a dry mouth, constipation, and blurred vision, and demonstrate variable efficacy across patient populations [6]. Furthermore, female bladder dysfunction arises from multiple etiological factors, including parturition, menopause, pelvic floor muscle weakness, and hormonal changes [7]. These multifaceted pathologies necessitate the development of novel therapeutic strategies that move beyond mere symptomatic relief to address underlying tissue remodeling, such as inflammation and fibrosis.

*Quisqualis indica* is a traditional medicinal herb used in Korean medicine for its anthelmintic properties, as well as for clearing accumulations and strengthening spleen and gastric functions [8]. Pharmacological studies have revealed that *Quisqualis indica* extract (QIE) offers various bioactive properties, including anti-inflammatory [9], antioxidant [10], and antibacterial [11] activities. Recent studies have reported the potential efficacy of *Quisqualis indica* in managing urogenital disorders, with preclinical models of benign prostatic hyperplasia [12,13,14] and clinical improvements in male patients with lower urinary tract symptoms (LUTS) [15]. There is growing interest in the therapeutic potential of *Quisqualis indica* for bladder function regulation, yet research on female bladder dysfunction has been limited.

The present study used an animal model comprising partial bladder outlet obstruction (pBOO) induced in female rats to investigate the effects of QIE on female bladder dysfunction. Following pBOO induction, rats were treated with QIE for 7 weeks, and their voiding patterns, bladder function, and histological/molecular markers of inflammation and fibrosis were evaluated. Furthermore, we assessed the direct contraction-relieving effects of QIE using isolated bladder detrusor strips. Our findings demonstrate that QIE alleviates bladder inflammation and fibrosis in a bladder dysfunction model of female rats, which is mechanistically associated with the modulation of muscarinic-mediated bladder contractility.

## 2. Results

### 2.1. Quality Control and Standardization of QIE

**Figure 1:**
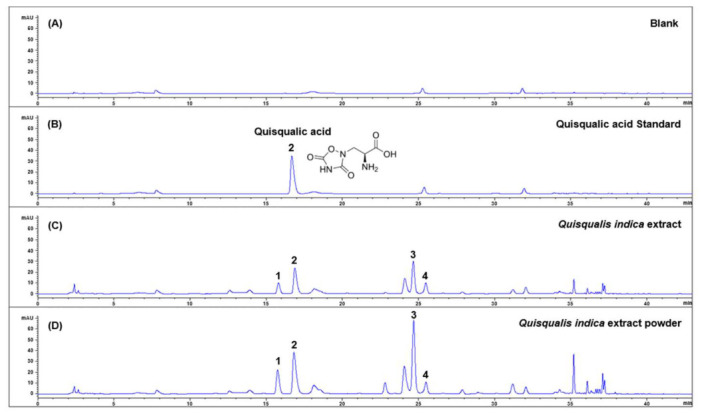
HPLC chromatogram. (**A**) Blank, (**B**) Quisqualic acid (QA) standard, (**C**) liquid EtOH extract of *Quisqualis indica* (before spray-drying), (**D**) *Quisqualis indica* extract (QIE) powder (after spray-drying). 1, asparagine; 2, QA; 3, arginine; 4, glutamic acid.

The chromatographic profiles of QIE before and after the spray-drying process showed no substantial differences in peak patterns, indicating that the major phytochemical constituents were well-preserved during manufacturing (Figure 1). The HPLC chromatograms were utilized primarily to ensure the quality control and batch-to-batch consistency of the standardized extract. Notably, in addition to the marker compound, peaks corresponding to previously identified constituents such as asparagine, arginine, and glutamic acid were also consistently observed following the spray-drying process [14].

To determine the content of quisqualic acid (QA) in the QIE dried powder, quantitative analysis was performed using HPLC. The calibration curve was constructed by plotting the peak area against six concentrations of the QA standard, ranging from 0.0125 to 0.4 mg/mL, using least-squares regression analysis. The calibration curve exhibited good linearity with a correlation coefficient (r^2^) greater than 0.99. Based on this curve, the content of QA in the final QIE powder was determined to be 10.205 ± 0.120 mg/g. These results confirmed that the final product met the predefined specification (1% QA), demonstrating the chemical consistency and robustness of the extract used in this study.

### 2.2. QIE Improved pBOO-Induced Urinary Dysfunction

**Figure 2:**
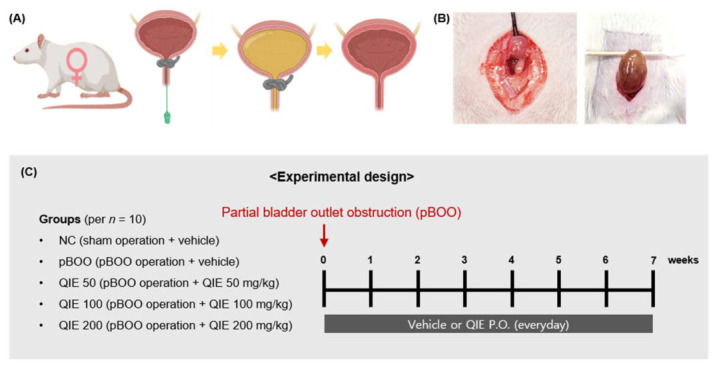
Schematic of partial bladder outlet obstruction (pBOO) modeling and QIE administration. (**A**) In female SD rats, a partial urethral obstruction was induced using a 22-gauge vascular catheter to establish a pBOO model. (**B**) Representative photographs of the urinary bladders of rats that underwent the sham operation (**left**) and rats in the pBOO group that underwent the pBOO operation (**right**). (**C**) Experiment timeline and group allocation for QIE administration following pBOO surgery The pBOO surgery was performed at week 0 (red arrow), and the drug was administered daily for 7 weeks.

**Figure 3:**
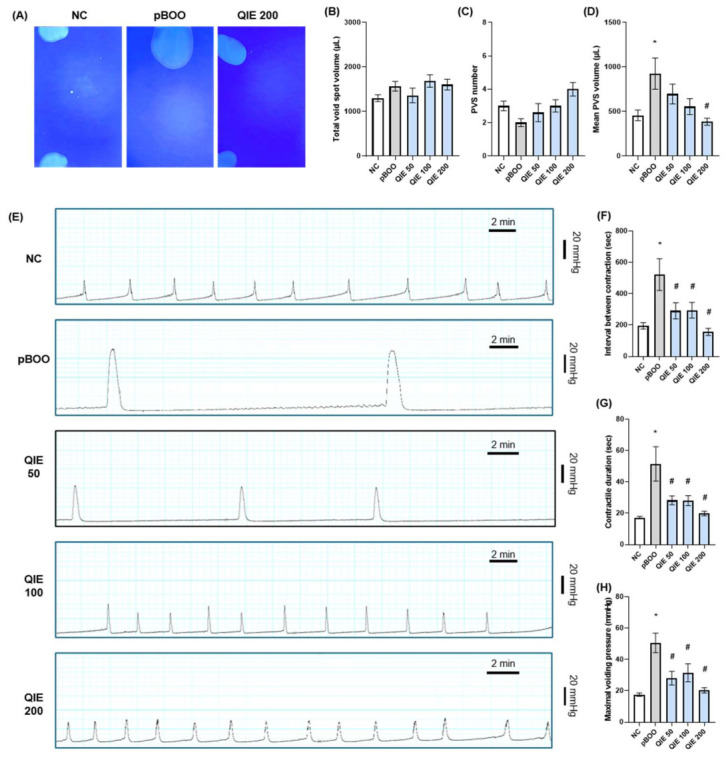
Void spot assay on day 48 after pBOO surgery and cystometry performed on day 49 after pBOO model surgery. (**A**) Absorbent paper that recorded spontaneous urination over a 4 h period and was photographed under UV light. (**B**) Total void spot volume obtained by converting the void spot size into urine volume. (**C**) Number of primary void spots (PVSs) larger than 20 µL. (**D**) The mean PVS volume was calculated by dividing the total PVS volume by the number of PVSs. The pBOO group exhibited a significantly higher mean PVS volume compared to the NC group (* *p* < 0.05 for NC vs. pBOO), and QIE 200 showed a significantly reduced mean PVS volume compared to the pBOO group (# *p* < 0.05 for pBOO vs. QIE 200). Sample size: NC (*n* = 9), pBOO (*n* = 9), QIE 50 (*n* = 10), QIE 100 (*n* = 10), QIE 200 (*n* = 8). Data are shown as mean ± SEM. (**E**) Representative cystometry results for each group. The time scales vary; refer to the scale bar on the right side of each panel. (**F**) Inter-contraction interval, defined as the time between the peak pressure points of two consecutive bladder contractions. (**G**) Contraction duration, defined as the time between reaching threshold pressure and returning to baseline pressure. (**H**) Maximal voiding pressure, defined as the highest pressure generated by the bladder muscle during urination (*n* = 10 per group). * *p* < 0.05 compared to NC; # *p* < 0.05 compared to pBOO. Data are presented as mean ± SEM.

To investigate whether QIEs ameliorate bladder voiding dysfunction, the pBOO model was induced by partially ligating the proximal urethra, and QIE was administered daily for 7 weeks at specified concentrations (Figure 2). After 48 days of oral administration, a void spot assay was conducted. The results obtained after allowing the rats to urinate spontaneously for 4 h are presented in Figure 3. The total void spot volume did not differ significantly across all experimental groups (Figure 3B). Although the number of primary void spots decreased in the pBOO group, this difference was not statistically significant (Figure 3C). Notably, the pBOO group exhibited larger void spots compared to other groups, with their mean primary void spot (PVS) volume significantly increased relative to the NC group (Figure 3D). In the QIE 200 group, the void spot size approximated that of the NC group, and the mean PVS volume was reduced compared to that of the pBOO group.

On day 49, cystometry was performed to measure intravesical pressure dynamics. In pBOO, strong detrusor muscle contractions are required to urinate due to bladder outlet obstruction. The cystometry showed that the pBOO group had a longer interval between contractions and a longer contractile duration (Figure 3F,G). The maximal voiding pressure also increased significantly (Figure 3H), indicating that the bladder underwent functional changes to overcome the increased outlet resistance. In QIE 50, 100, and 200 administration groups, the contraction interval and duration and the maximal voiding pressure were significantly reduced compared to the pBOO group. Cystometric parameters in the QIE 200 group were restored to levels comparable to those in the NC group.

### 2.3. QIE Mitigated pBOO-Induced Structural Changes in the Bladder

**Figure 4:**
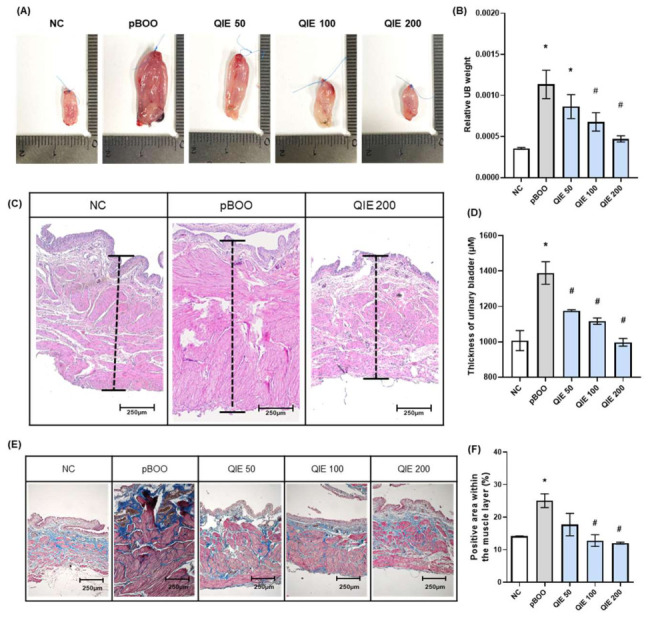
Macroscopic and histological evaluation of the bladder on day 49 after pBOO surgery. (**A**) Comparison of bladder size following excision from euthanized animals. (**B**) Relative bladder weight determined by dividing bladder weight by body weight (*n* = 10 per group). (**C**) H&E-stained histological sections of the bladders for evaluating bladder wall thickness. The dashed line indicates the thickness of the bladder wall. (**D**) Quantitative comparison of bladder thickness of the bladder wall between groups following H&E staining (*n* = 3 per group). (**E**) Masson’s trichrome staining in bladders to evaluate fibrosis. Red: muscle/cytoplasm. Blue: collagen. (**F**) Quantitative analysis of the percentage of collagen area in the muscularis layer (%) (*n* = 3 per group). * *p* < 0.05 compared to NC; # *p* < 0.05 compared to pBOO. Data are presented as mean ± SEM.

The pBOO model with proximal urethral ligation is characterized by chronic bladder outlet obstruction, which requires strong bladder muscle contractions, leading to thickening of the bladder wall and bladder enlargement. Consistent with this pathophysiology, the pBOO group showed an increase in bladder size and weight compared to the NC group. In contrast, the QIE 100 and 200 groups exhibited a significant reduction in bladder weight compared to the pBOO group (Figure 4A,B). H&E staining was performed to evaluate histological changes in the bladder wall. The pBOO group demonstrated significantly increased bladder wall thickness, while all QIE-treated groups showed a decrease in thickness (Figure 4C,D). Masson’s trichrome staining was additionally performed to assess fibrosis resulting from bladder obstruction. In pBOO specimens, collagen, which stains dark blue, was extensively deposited in the suburothelial region. Conversely, collagen deposition was markedly attenuated in the QIE-treated group (Figure 4E). Quantitative analysis was performed to determine the percentage of collagen area relative to the total tissue area within the muscularis layer. In the pBOO group, the proportion of collagen area was significantly increased compared to the NC group. Conversely, this percentage was significantly reduced in both the QIE 100 and QIE 200 groups compared to the pBOO group, demonstrating that QIE treatment effectively inhibits the excessive accumulation of collagen induced by bladder outlet obstruction (Figure 4F).

### 2.4. QIE Improved pBOO-Induced Bladder Inflammation and Molecular Changes

**Figure 5:**
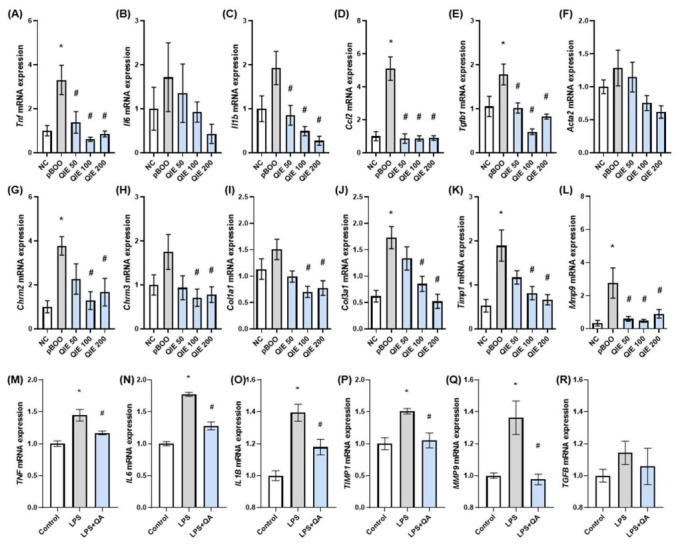
Evaluation of inflammation and molecular changes in the bladder and human bladder epithelial cell lines via quantitative RT-PCR. (**A**) *Tnf* (TNF-α), (**B**) *Il6* (IL-6), (**C**) *Il1b* (IL-1β), (**D**) *Ccl2* (MCP-1), (**E**) *Tgfb1* (TGF-β), (**F**) *Acta2* (α-SMA), (**G**) *Chrm2* (M2 receptor), (**H**) *Chrm3* (M3 receptor) (**I**) *Col1a1* (Collagen type I α1), (**J**) *Col3a1* (Collagen type III α1), (**K**) *Timp1* (TIMP-1), and (**L**) *Mmp9* (MMP-9) mRNA expression normalized to GAPDH. Sample sizes: NC (*n* = 5), pBOO (*n* = 5), QIE 50 (*n* = 6), QIE 100 (*n* = 6), and QIE 200 (*n* = 6). * *p* < 0.05 compared to NC, # *p* < 0.05 compared to pBOO. Data are presented as mean ± SEM. (**M**–**R**) 5637 cells were pre-treated with QA (1 μM) for 2 h and then stimulated with LPS (10 μg/mL) for 24 h. The mRNA expression levels of (**M**) *TNF* (TNF-α), (**N**) *IL6* (IL-6), (**O**) *IL1B* (IL-1β), (**P**) *TIMP1* (TIMP1), (**Q**) *MMP9* (MMP9), and (**R**) *TGFB* (TGF-β) were determined by quantitative RT-PCR. Data are presented as mean ± SEM. (*n* = 4) * *p* < 0.05 compared to Control, # *p* < 0.05 compared to LPS.

TNF-α, IL-6, IL-1β, and MCP-1 are cytokines or chemokines that mediate bladder inflammatory responses, and increased expression of these factors induces pathological changes such as bladder dysfunction and chronic tissue damage. In the pBOO-induced group in this study, the expression of these genes was elevated compared to the control group, with TNF-α and MCP-1 showing particularly significant increases in the pBOO group. In contrast, the expression of these genes was reduced in QIE-treated groups. Specifically, TNF-α, IL-1β, and MCP-1 were significantly decreased in all QIE-treated groups compared to the pBOO group (Figure 5A–D).

To evaluate bladder fibrosis, the expression of TGF-β and α-SMA was assessed. TGF-β and α-SMA are representative genes upregulated under chronic stress induced by bladder outlet obstruction and are key mediators of bladder fibrosis. TGF-β expression was significantly increased in the pBOO group compared to the NC group and decreased in QIE administration groups. No significant difference in α-SMA expression was observed among all groups (Figure 5E,F).

M_2_ and M_3_ receptors are the primary muscarinic acetylcholine receptors present in the detrusor muscle. The M_3_ receptor is known to be directly responsible for bladder contraction, while the M_2_ receptor is known to indirectly facilitate contraction. Expression of these receptors can be altered in association with pathological changes in the bladder. In this study, M_3_ expression was significantly increased in the pBOO group compared to the NC group, whereas M_2_ expression showed a non-significant trend toward increased expression. In the QIE 100 and 200 treatment groups, both M_2_ and M_3_ expressions were significantly reduced compared to the pBOO group (Figure 5G,H).

Furthermore, the expression of genes associated with extracellular matrix (ECM) remodeling was evaluated (Figure 5I–L). In the pBOO-induced group, the expression of Collagen type III α1 was significantly upregulated compared to the NC group, while Collagen type I α1 showed an increasing trend without reaching statistical significance. Additionally, the expression of TIMP-1 and MMP-9, key regulators of ECM stabilization and degradation, significantly increased following pBOO induction. In contrast, QIE treatment effectively suppressed the expression of all these fibrosis-related factors; specifically, the expression levels of Collagen type I α1, Collagen type III α1, TIMP-1, and MMP-9 were significantly reduced in both the QIE 100 and 200 groups compared with the pBOO group.

### 2.5. QA Attenuates Inflammatory and Remodeling Gene Expression in 5637 Cells

To investigate the anti-inflammatory and protective effects of QA, which was previously utilized as a standard compound for QIE standardization, we evaluated the mRNA expression levels of pro-inflammatory cytokines (TNF-α, IL-6, IL-1β) and tissue remodeling-related factors (TIMP1, MMP9, TGF-β) against lipopolysaccharide (LPS)-induced damage in 5637 human bladder epithelial cells (Figure 5M–R). For this study, the cells were divided into three groups: the untreated control group, the LPS group (stimulated with 10 μg/mL LPS for 24 h), and the LPS + QA group (pre-treated with 1 μM QA for 2 h prior to the 24 h LPS exposure).

Exposure to LPS significantly upregulated the mRNA expression of TNF, IL6, IL1B, TIMP1, and MMP9 compared to the untreated control group (*p* < 0.05). However, pre-treatment with QA effectively suppressed the LPS-induced elevation of these inflammatory and remodeling markers (TNF-α, IL-6, IL-1β, TIMP1, and MMP9), showing a statistically significant inhibitory effect (*p* < 0.05). Meanwhile, the mRNA expression of TGF-β showed no statistically significant differences among the control, LPS, and LPS + QA groups.

### 2.6. QIE Alleviated the Augmented Muscarinic Contraction of Bladder Smooth Muscle in pBOO

**Figure 6:**
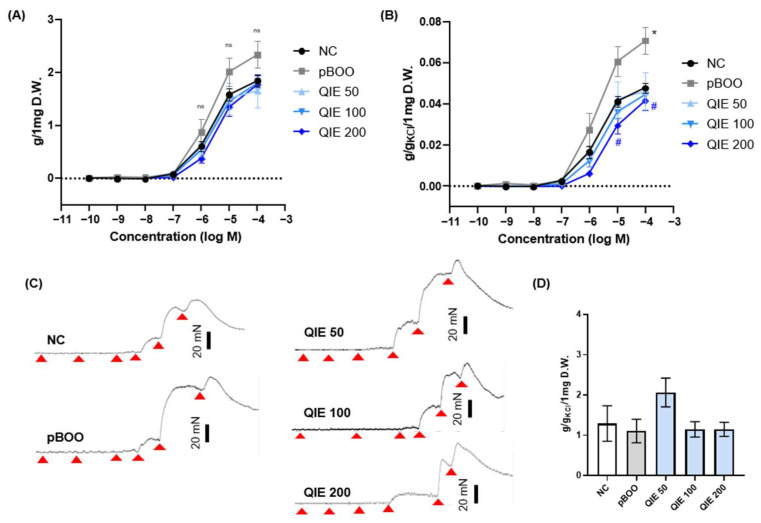
Evaluation of muscarinic and purinergic contraction of the bladder smooth muscle. (**A**,**B**) Thirty minutes following pre-treatment with QIE or vehicle at each concentration, carbachol was applied to obtain the concentration–response curve. (**A**) Comparison of the contraction force per unit weight. (**B**) Comparison of contraction force per unit tissue weight following normalization to contraction magnitude induced by 60 mM KCl (*n* = 12 per group). (**C**,**D**) 30 min after pre-treating with QIE or vehicle at each concentration, purinergic contraction was assessed by treatment with 100 μM ATP-Na. (**C**) Representative figure for each treatment group. (Red arrowheads) ATP was administered at increasing concentrations to establish a dose-response relationship. (**D**) Comparison of contraction force per unit tissue weight following normalization to contraction magnitude induced by 60 mM KCl. Sample sizes: NC (*n* = 7), pBOO (*n* = 5), QIE 50 (*n* = 5), QIE 100 (*n* = 7), and QIE 200 (*n* = 8). ns: not significant compared to NC, * *p* < 0.05 compared to NC, and # *p* < 0.05 compared to pBOO. Data are presented as mean ± SEM.

The pBOO model was induced, and at week 7, the bladder was excised to generate a longitudinal bladder muscle strip containing the urothelium for contractility assessment (Figure 6). To compare the muscarinic contractility of the bladder smooth muscle, the muscarinic receptor agonist carbachol was administered in an accumulative manner from 10^−10^ M to 10^−4^ M to obtain a concentration–response curve. When comparing the contraction force per unit weight, relative contraction force showed an increasing trend in the pBOO group; however, this difference did not achieve statistical significance (Figure 6A). However, upon normalization to the contraction magnitude induced by 60 mM KCl and the subsequent comparison of contraction force, muscarinic contraction in pBOO was found to be significantly increased compared to the control group (Figure 6B). In the QIE 200 group, the enhanced muscarinic contraction induced by pBOO was significantly attenuated. Purinergic contraction was evaluated by applying 100 μM ATP, a purinergic agonist. ATP-induced contraction was observed in all experimental groups (Figure 6C,D). After adjusting for contraction magnitude induced by 60 mM KCl, the pBOO group exhibited reduced contraction force compared to the NC group, but this difference was not statistically significant. Similarly, no significant differences were observed in QIE-treated groups.

## 3. Discussion

In this study, we examined the effects of QIE on bladder dysfunction to evaluate its potential utility as a therapeutic candidate. For this purpose, animal models were used in which bladder dysfunction was induced in female rats via pBOO. The pBOO animal model undergoes sequential pathological stages—hypertrophy, a compensated phase, and a decompensated phase—according to disease progression, thereby manifesting characteristics of both an overactive bladder (OAB) and underactive bladder (UAB) [16,17,18]. In the early stages of pBOO, the bladder undergoes a hypertrophic phase to overcome the resistance imposed by lower urinary tract obstruction. Compensatory changes include hypertrophy of the detrusor muscle and increased expression of M_3_ muscarinic receptors to augment contractile force, thereby increasing pressure to expel urine [19]. The decompensatory stage is characterized by persistent chronic obstruction and overload, culminating in a deterioration in bladder function. This stage is marked by bladder fibrosis, characterized by the replacement of bladder muscle cells with fibrous tissue such as collagen and increased gene expression of TGF-β, α-SMA, and related mediators [20,21]. The characteristics of the pBOO model have been confirmed in multiple studies and are observed not only in animal models but also in clinically defined human bladder outlet obstruction (BOO) [16,21]. Rat models of bladder dysfunction using pBOO are also commonly used [17]. In rat models, the compensatory phase is observed during the early stages of obstruction and is characterized by hypertrophy of the bladder muscle and increased contractility. Subsequently, obstruction models persisting for 4–8 weeks or longer exhibit fibrosis of the bladder and decreased contractility, demonstrating features of the decompensated phase [22,23]. However, depending on the experimental design, these phases may vary significantly and can manifest in a mixed pattern [17,24]. In the pBOO model used over 7 weeks in this study, characteristics of both the compensatory phase, including bladder wall hypertrophy and enhanced bladder contractility, and the decompensatory phase, including an abnormally enlarged, overfilled bladder and bladder fibrosis, were observed.

Within this pBOO model, the present study investigated the effect of QIE in the amelioration of bladder dysfunction. *Quisqualis indica* is a vine-like climbing plant traditionally regarded as a medicinal herb and primarily used in anthelmintic treatments, for diarrhea, and for abdominal pain [8]. *Quisqualis indica* exhibits antibacterial [11], antiviral [25], and anthelmintic [26] effects, in addition to anti-inflammatory [9] and antioxidant [10] effects. Previous studies have demonstrated that *Quisqualis indica* alleviates prostatic enlargement and lower urinary tract symptoms in BPH animal models [12,13,14]. A clinical trial involving male LUTS patients has similarly demonstrated that *Quisqualis indica* extract also improved symptoms of urinary urgency and frequent urination [15]. However, there are limited studies examining the effects of *Quisqualis indica* in female lower urinary tract disorders. Therefore, the present study aimed to investigate the effects of *Quisqualis indica* extract in female lower urinary tract disorders by inducing pBOO in female rats. We employed the pBOO model, despite its higher clinical prevalence in males, to maintain a consistent experimental framework for comparison with prior male-based studies. Furthermore, by utilizing an identical standardized extract to that used in previous male-based studies [14], we were able to suggest that the therapeutic potential of the extract is not merely secondary to a reduction in prostate size but extends to the direct protection of the bladder wall in females.

Following the induction of pBOO in female rats, QIE was administered daily for 7 weeks. Our results confirmed that QIE effectively improved urinary dysfunction and simultaneously alleviated pathological changes, including bladder inflammation and fibrosis. The QIE-treated group demonstrated improved voiding patterns, including contraction intervals and intravesical pressure, compared to the untreated pBOO group. With saline infused at a constant rate, ICI served as a functional surrogate for bladder capacity. While residual volume was not measured, the significant changes in ICI, MVP, and contraction duration sufficiently demonstrated QIE’s efficacy in recovering voiding efficiency and ameliorating obstructive pathology. Histological analysis of bladder tissue revealed significant reductions in muscle hypertrophy and collagen deposition. These findings corresponded with a reduced expression of inflammatory factors such as TNF-α, IL-6, IL-1β, and MCP-1, as well as fibrosis-related genes. QIE treatment was accompanied by a reduction in the pBOO-induced upregulation of TGF-β, Collagen type III α1, TIMP-1, and MMP-9, while also showing an inhibitory trend toward Collagen type I α1 expression. To further elucidate whether these protective actions are directly mediated by the active constituents of QIE at the cellular level, we evaluated the effects of QA, a major constituent of QIE, in 5637 human bladder epithelial cells. While these in vitro findings provide valuable insights into the bioactivity of QA, the therapeutic benefits observed in our in vivo experiments likely reflect the integrative actions of the various constituents within the QIE mixture. Consistent with this holistic perspective, pre-treatment with QA significantly suppressed the LPS-induced upregulation of pro-inflammatory cytokines (TNF-α, IL-6 and IL-1β) and tissue remodeling factors (TIMP1 and MMP9). Furthermore, QIE reduced the elevated expression of muscarinic receptors (M_2_ and M_3_) in the pBOO model and directly alleviated the contractility of bladder smooth muscle strips induced by muscarinic agonists, suggesting a potential association between the therapeutic effects of QIE and the modulation of muscarinic pathways. Further direct target-specific validation is required to fully elucidate the molecular interactions involved.

In bladder disease models, pathological conditions are frequently accompanied by an increased expression of muscarinic receptors along with excessive contractile activity of the bladder muscle [23,27,28]. Therefore, current therapeutic approaches to bladder dysfunction have primarily focused on inhibiting receptor function [29]. In contrast, QIE presents a therapeutic strategy that simultaneously alleviates not only urinary dysfunction but also chronic inflammation and fibrosis of the bladder. By reducing the expression of receptors associated with bladder dysfunction, QIE may contribute to the normalization of excessive muscarinic contractility, offering a potential mechanism to address the underlying pathophysiology. Interestingly, the potential of *Quisqualis indica* in modulating muscarinic pathways is further supported by recent in silico evidence. Studies have indicated that several bioactive compounds found in *Quisqualis indica* leaf extracts, including beta-sitosterol, quercetin, and lupeol, demonstrate favorable binding affinities with the M2 muscarinic receptor [30]. Future studies employing direct binding assays and functional validation are essential to confirm these molecular interactions and their pharmacological significance. Moreover, *Quisqualis indica* is a natural product widely used in traditional medicine, offering the advantage of minimal adverse effects. In this study, QIE was administered daily for 7 weeks without any evidence of weight changes or organ dysfunction. Existing preclinical and clinical trial data support the potential human application of *Quisqualis indica* [15,31]. Prior toxicological assessments established a no-observed-adverse-effect level (NOAEL) for QIE exceeding 2000 mg/kg/day, with no treatment-related systemic or genotoxic effects. These findings confirm that the doses employed in the current study remain well within a validated safety range [31].

Despite the significant findings regarding the therapeutic potential of QIE, this study has several limitations. First, this study utilized HPLC analysis to ensure the quality control and batch-to-batch consistency of the standardized extract. However, it is important to acknowledge that this approach provides a targeted quality assessment rather than an exhaustive phytochemical characterization. While the stability of the marker compound, QA, and the consistent presence of major constituents such as asparagine, arginine, and glutamic acid have been confirmed as previously reported, the entire phytochemical profile remains to be verified. Future work should employ comprehensive phytochemical profiling to fully characterize the impact of spray-drying on the extract’s composition. Second, the absence of a maltodextrin-only control group is a methodological constraint. While existing evidence suggests that maltodextrin does not modulate muscarinic receptors or fibrotic signaling pathways [32], its potential influence on gut microbiota [33,34] necessitates the inclusion of a maltodextrin-matched vehicle control in future studies to definitively isolate the effects of the active extract from those of the carrier. We acknowledge that our reliance on a PBS-only control, which lacks the carrier, prevents us from fully excluding the potential contribution of maltodextrin to the observed therapeutic outcomes. Finally, while our study observed significant improvements in inflammatory and muscarinic signals, it is important to interpret these mechanistic findings with caution. Although QA, a major constituent of QIE, exhibited potent anti-inflammatory and anti-fibrotic effects in vitro, the therapeutic efficacy observed in vivo should be considered as the holistic outcome of the entire extract. The potential synergistic interactions between QA and other constituents in the complex mixture of QIE warrant further investigation.

In conclusion, this study demonstrated that QIE alleviates bladder inflammation and fibrosis in a female rat model of pBOO. *Quisqualis indica* has been utilized primarily for male lower urinary tract disorders; however, our findings suggest its therapeutic potential for female populations as well. Further rigorous research, including the isolation of active ingredients, receptor-binding assays, and direct target-specific validation, is essential to definitively confirm the pharmacological precision and elucidate the underlying mechanisms of QIE.

## 4. Materials and Methods

### 4.1. Animals

Seven-week-old female Sprague Dawley (SD) rats were purchased from Orient Bio (Seongnam, Republic of Korea) and acclimated for one week in the animal facility prior to experimental use. The facility had a maintained temperature of 23 ± 1 °C, humidity of 40–60%, and ventilation of 12–15 air changes per hour. A 12 h light–dark cycle was maintained at 200–300 lux, and all light was blocked. Food and water were provided in sufficient quantities without restriction. All experimental protocols were approved by the Chungnam National University Institutional Animal Care and Use Committee (approval number: 202404A-CNU-079).

### 4.2. Preparation of pBOO Model

The pBOO model, created by partially ligating the proximal urethra (approximately 30–50%), was selected as an animal model to subject the bladder to sustained mechanical stress. Female SD rats weighing 250–300 g were anesthetized using 2–3% isoflurane. After anesthesia stabilized, a midline incision was made in the lower abdomen to expose the bladder. The surrounding adipose tissue was carefully dissected, and a 22-gauge vascular catheter was inserted through the urethra and up through the bladder. The urethra and vagina were carefully separated at the level of the bladder neck, and the urethra was ligated three times using a 4-0 silk suture with surgeon’s knots. The catheter was then removed. The abdominal wall and skin were closed in layers, and post-operative analgesia and antimicrobial prophylaxis were provided via the administration of meloxicam (1 mg/kg) and enrofloxacin (1 mg/kg). Sham-operated control animals underwent identical surgical procedures excluding urethral ligation.

### 4.3. QIE Standard Solution Preparation

*Quisqualis indica* was purchased from a domestic herbal market (Ansan, Republic of Korea) and authenticated by Dr. Yeon before being deposited in the HUONS Research Center Herbarium (specimen number HU033/QQA23025, Ansan, Republic of Korea). The extraction conditions were maintained based on a previously optimized protocol [14]. Dried *Quisqualis indica* was homogenized into a fine powder (50 kg), then reflux-extracted with 500 L of 70% ethanol at 80 °C for 6 h. The extract was concentrated under vacuum at 60 °C until the organic solvent was completely removed. The extraction yield was determined to be 41.4% relative to the raw material, and a total of 20.7 kg of extract was ultimately obtained. The drug–extract ratio (DER) was calculated to be 2.42:1. To ensure the stability of the active compounds, the concentrate was mixed with maltodextrin—a pharmaceutical excipient—in a 1:1 mass ratio (*w*/*w*) relative to the total soluble solids content. The mixture was then subjected to spray drying using a spray dryer (ODA-25, SeoGang Engineering, Cheonan, Republic of Korea) with the following optimized parameters: an inlet temperature of 170–180 °C, an outlet temperature of 80–90 °C, and a feed rate of 10 mL/min.

The final product (QIE) was chemically standardized via a validated HPLC method as previously described [14], with specific modifications detailed below. Chromatographic analysis was performed using an Agilent HPLC system equipped with an Eclipse Plus C18 column (4.6 × 250 mm, 5 μm) maintained at 30 °C. The mobile phase consisted of solvent A (0.2 M acetic acid, 0.01 M sodium acetate, and water; 51:49:900, *v*/*v*) and solvent B (methanol). A gradient elution was applied as follows: 0–5 min, 20% B; 5–14 min, 20–30% B; 14–30 min, 30–50% B; 30–35 min, 50–100% B; 35–38 min, 100% B; 38–40 min, 100–20% B; and 40–43 min, 20% B, followed by a 3 min post-run re-equilibration. The flow rate was set at 1.0 mL/min. For analysis, 10 μL of the sample was mixed with 50 μL of phthaldialdehyde (OPA) reagent, and an injection volume of 60 μL was used. The effluent was monitored at a detection wavelength of 338 nm with a reference wavelength of 390.2 nm. A representative chromatogram of the QIE is presented in Figure 2. The administered QIE product was standardized to ensure a consistent QA content of 1% for pharmacological reliability.

### 4.4. QIE Administration

Before pBOO surgery, a total of 50 female rats were randomly assigned to five experimental groups (*n* = 10 per group): (1) NC: sham-operated group receiving vehicle; (2) pBOO: pBOO surgery group receiving vehicle; (3) QIE 50: pBOO surgery group receiving 50 mg/kg QIE; (4) QIE 100: pBOO surgery group receiving 100 mg/kg QIE; (5) QIE 200: pBOO surgery group receiving 200 mg/kg QIE. QIE dosages refer to the total weight of the final standardized formulation (1:1 *w*/*w* with maltodextrin). The sample size was determined based on previous studies [17,24]. Phosphate-buffered saline (PBS) was used as the vehicle for both the control and treatment groups. QIE was freshly prepared as a suspension in PBS immediately prior to each administration to maintain its pharmacological consistency and stability. All animals received either the vehicle or QIE once daily for 7 weeks (49 days) using a standard stainless steel feeding needle (oral zonde), and animals were euthanized on the final day of administration.

### 4.5. Void Spot Assay

Void spot assays were performed on day 48 post-surgery to assess spontaneous voiding behavior. Clean rat cages were prepared with absorbent filter paper (3 mm Whatman CHR, Cytiva, Marlborough, MA, USA) placed on the cage floor. To prevent fecal contamination, filter paper was covered with a stainless mesh. Food and water were restricted, and animals were permitted to move freely in a darkened room to encourage spontaneous urination. After 4 h, the animals were removed, and the filter papers were collected. Voiding spots were photographed under 350 nm ultraviolet illumination, and voiding frequency and volume were evaluated. By dropping serial volumes of saline onto the same filter paper, we plotted an area–volume curve and calculated the void spot size as the urine volume. The primary void spots (PVSs) were defined as spots corresponding to volumes ≥ 20 µL. Individual spots were manually traced along their perimeters, which enabled the distinct identification of boundaries even in cases of partial overlap. Certain samples were excluded due to unquantifiable artifacts, including severe fecal contamination, merged urine stains, or physical damage (e.g., gnawing) to the filter paper.

### 4.6. Cystometry

On day 49 post-pBOO surgery, cystometric evaluation was performed to assess the bladder function improvement effect of QIE. Animals were anesthetized with an intraperitoneal injection of urethane (1.2 g/Kg diluted in physiological saline) and stabilized for 30–60 min. Following stabilization, animals were placed on a 37 °C temperature-controlled surgical platform, and the surgical site was shaved and disinfected. A lower abdominal midline incision was made to expose the bladder, and the dome of the exposed bladder was incised using an 18-gauge needle. A polyethylene catheter (PE-50, ADInstruments, Dunedin, New Zealand) was inserted into the incised bladder and was fixed using a purse-string suture technique with non-absorbable nylon suture (6-0, Ethicon, Raritan, NJ, USA). The PE catheter was connected using a 3-way valve to both a pressure transducer (Harvard Apparatus, Holliston, MA, USA) and a syringe pump (NE-1000, New Era Pump Systems, Inc., Farmingdale, NY, USA). Following a 30-min stabilization period during which physiological saline was infused at 5 mL/h, bladder voiding functions were recorded. Intravesical pressure changes were recorded using a PowerLab 4/26 (ADInstruments) data acquisition system and analyzed with LabChart software (version 8, ADInstruments). There were no animal deaths or experimental failures during the study; therefore, no data points were excluded from the analysis.

### 4.7. Histological Analysis

After cystometry, the bladder urine was drained via catheterization, and the catheter was carefully removed. Bladders were excised, incised along the median axis, and fixed in 10% buffered formalin solution for ≥24 h. The fixed bladder tissues were washed with tap water to remove residual formalin and then immersed in a series of ethanol and xylene steps and embedded in paraffin to generate paraffin blocks. The blocks were sectioned at 4 µm thickness and mounted on glass slides. The sections were then deparaffinized in xylene and stained with Harris Hematoxylin Solution (#H08-500R, TissuePro Technology, Gainesville, FL, USA) and Eosin Y Solution (#EY07-500R, TissuePro Technology) for H&E staining. Mounting medium (#H-5700-60, VectaMount Express, Newark, CA, USA) was applied, and dried sections were digitized at 400× magnification using a Digital Slide Scanner (Motic^®^ easy scan Pro-6, Hong Kong, China). Histological evaluation of scanned bladders was performed by a researcher unaware of group assignments using ImageJ software (version 46a; NIH, Bethesda, MD, USA). Bladder wall thickness was measured at five randomly selected areas per specimen, and the average value was obtained.

### 4.8. Masson’s Trichrome Staining

Bladders were excised, incised medially, and fixed in 10% buffered formalin solution for at least 24 h prior to paraffin embedding. Blocks were sectioned at 4 µm thickness and mounted on glass slides. To evaluate fibrosis of the bladder wall, Masson’s trichrome staining was performed using a commercial kit (#BAQ085, GBioscience, St. Louis, MO, USA) according to the manufacturer’s principles. Briefly, tissues were fixed in Bouin’s reagent at 60 °C for 1 h, rinsed with running water, and cooled for 10 min. Sections were treated with Weigert’s reagent for 5 min, rinsed, stained with Briebrich Scarlet for 15 min, and decolorized in phosphotungstic phosphomolybdic acid for 15 min. Following staining with aniline blue reagent for 10 min and rinsing, the sections were treated with 1% glacial acetic acid for 5 min, dehydrated through graded ethanol, and mounted using mounting medium (#H-5700-60, VectaMount Express). Slides were scanned at 400× magnification using a Digital Slide Scanner (Motic^®^ easy scan Pro-6).

### 4.9. Quantitative RT-PCR of Bladder Tissue

After euthanasia, bladders were harvested and stored at −80 °C until analysis. Tissues were homogenized using the BIOPERP-24R Bead Homogenizer (Bioand Corporation, Guri, Republic of Korea), and total RNA was extracted using a commercial kit (#SG-RN-TRN-L, SmartGENE, Daejeon, Republic of Korea) according to the manufacturer’s instructions. cDNA was synthesized from 1 µg of RNA using the cDNA Synthesis kit (#SG-cDNAC100, SmartGENE). The synthesized cDNA was diluted 10-fold in RNase-free D.W. and subjected to quantitative PCR using SYBR Green Q-PCR Master Mix (#SG-SYBR-ROXH, SmartGENE). The primer sequences used for q-PCR were as follows: *Gapdh* (GAPDH) forward primer: 5′-ACA GCA ACA GGG TGT GGA C-3′, reverse primer: 5′-TTT GAG GGT GCA GCG AAC TT-3′, *Tnf* (TNF-α) forward primer: 5′-GTC TGT GCC TCA GCC TCT TC-3′, reverse primer: 5′-CCC ATT TGG GAA CTT CAC CT-3′, *Il6* (IL-6) forward primer: 5′-CTT GGG ACT GAT GTT GTT GA-3′, reverse primer: 5′-CTC TGA ATG ACT CTG GCT TT-3′, *Il1b* (IL-1β) forward primer: 5′-ATG GCA ACT GCC CTG AAC T-3′, reverse primer: 5′-GTC ATC ATC CCA CGA GTC AC-3′, *Ccl2* (MCP-1) forward primer: 5′-GGC CTG TTG TTC ACA GTT GCT-3′, reverse primer: 5′-TCT CAC TTG GTT CTG GTC CAG-3′, *Tgfb1* (TGF-β) forward primer: 5′-AGG GCT CAA CTG CTG CAC-3′, reverse primer: 5′-GGG CCC CAG ACA GAA GTT-3′, *Acta2* (α-SMA) forward primer: 5′-ATA GAA CAC GGC ATC ATC ACC-3′, reverse primer: 5′-GGT CTC AAA CAT AAT CTG GGT-3′, *Chrm2* (M_2_) forward primer: 5′-CCA CTC CAG AGA TGA CAA CT-3′, reverse primer: 5′-GGC TAC AAC GTT CTG CTT T-3′, *Chrm3* (M_3_) forward primer: 5′-CAC GAA ACC TCT GAC CTA CCC-3′, reverse primer: 5′-TCT GAC CCG ACG ACC CAA CTA-3′, *Col1a1* (Collagen type I α1) forward primer: 5′-TCA AGA TGG TGG CCG TTA CT-3′, reverse primer: 5′-CAT CTT GAG GTC ACG GCA TG-3′, *Col3a1* (Collagen type III α1) forward primer: 5′-TGG GAT GCA ACT ACC TTG GT-3′, reverse primer: 5′-AGG TGT AGA AGG CTG TGG AC-3′, *Timp1* (TIMP-1) forward primer: 5′-CGC AGC GAG GAG GTT TCT CAT-3′, reverse primer: 5′-GGC AGT GAT GTG CAA ATT TCC-3′, *Mmp9* (MMP-9) forward primer: 5′-TCG GAT GGT TAT CGC TGG TG-3′, reverse primer: 5′-AAG ACG CAC ATC TCT CCT GC-3′.

### 4.10. 5637 Cell Cultures and Quantitative RT-PCR

The 5637 human bladder epithelial cell line was obtained from Korean Cell Line Bank (KCLB, Seoul, Republic of Korea). Cells were cultured in RPMI 1640 medium supplemented with 10% fetal bovine serum (FBS) and 1% penicillin/streptomycin at 37 °C in a humidified atmosphere containing 5% CO_2_. For the experiments, cells were seeded and pre-treated with 1 μM of quisqualic acid (QA) for 2 h prior to stimulation with 10 μg/mL of lipopolysaccharide (LPS) for an additional 24 h.

Total RNA was extracted from the treated 5637 cells using a commercial kit (#SG-RN-TRN-L, SmartGENE, Daejeon, Republic of Korea) according to the manufacturer’s instructions. cDNA was synthesized from 1 µg of RNA using the cDNA Synthesis kit (#SG-cDNAC100, SmartGENE). The synthesized cDNA was diluted 10-fold in RNase-free D.W. and subjected to quantitative PCR using SYBR Green Q-PCR Master Mix (#SG-SYBR-ROXH, SmartGENE). The primer sequences used for q-PCR were as follows: *GAPDH* (GAPDH) forward primer: 5′-GAA GGT GAA GGT CGG AGT C-3′, reverse primer: 5′-GAA GAT GGT GAT GGG ATT TC-3′, *TNF* (TNF-α) forward primer: 5′-CCT CTC TCT AAT CAG CCC TCT G-3′, reverse primer: 5′-GAG GAC CTG GGA GTA GAT GAG-3′, *IL6* (IL-6) forward primer: 5′-ACT CAC CTC TTC AGA ACG AAT TG-3′, reverse primer: 5′-CCA TCT TTG GAA GGT TCA GGT TG-3′, *IL1B* (IL-1β) forward primer: 5′-ATG ATG GCT TAT TAC AGT GGC AA-3′, reverse primer: 5′-GTC GGA GAT TCG TAG CTG GAT-3′, *TIMP1* (TIMP1) forward primer: 5′-TTT CCG ACC TCG TCA TCA GG-3′, reverse primer: 5′-ATG GTC TGG TTG ACT TCT GGT-3′, *MMP9* (MMP9) forward primer: 5′-TTG ACA GCG ACA AGA AGT GG-3′, reverse primer: 5′-GCC ATT CAC GTC GTC CTT AT-3′, *TGFB* (TGF-β) forward primer: 5′-CCT TTC CTG CTT CTC ATG GC-3′, reverse primer: 5′-TCC GTG GAG CTG AAG CAA TA-3′.

### 4.11. Bladder Smooth Muscle Strip Contractility

On day 49 post-pBOO surgery, animals were euthanized and the entire urinary system, including the bladder, uterus, and vagina, was excised at once and placed in physiological saline solution (PSS). The composition of PSS consisted of 120 mM NaCl, 2.5 mM CaCl_2_ (anhydrous), 1.0 mM MgCl_2_ (anhydrous), 11 mM glucose, 25 mM NaHCO_3_, 5.9 mM KCl, and 1.2 mM NaH_2_PO_4_·H_2_O. Under a stereomicroscope (Nikon SMZ-2T, Tokyo, Japan) in ice-cold PSS on a silicon-coated Petri dish, the surrounding tissue was carefully removed. A longitudinal incision was then made based on the bladder neck and spread open. Bladder smooth muscle strips approximately 10 mm in length were prepared by quartering the bladder without damaging the urothelium. The bladder strips were connected to the bottom of the organ bath, while the other end was connected to an isometric transducer (FT10, Grass instruments, West Warwick, RI, USA). Bladder tissue was continuously supplied with a 95% O_2_ and 5% CO_2_ mixed gas throughout the experiments to maintain physiological pH conditions.

Initially, contractile responses to 100 μM ATP (purinergic agonist) were compared across groups to assess purinergic contractility. To evaluate responsiveness to parasympathetic stimulation, carbachol (a muscarinic receptor agonist) was administered. Each contraction of QIE or vehicle was pre-treated for 30 min, followed by cumulative administration of carbachol at concentrations of 10^−10^, 10^−9^, 10^−8^, 10^−7^, 10^−6^, 10^−5^, and 10^−4^ M to generate a concentration-response curve. The contractions were detected using the PowerLab Data Acquisition system 4/26 (ADInstruments) and recorded and analyzed using LabChart software (version 8).

### 4.12. Statistics

All values in the results are expressed as mean ± standard error of the mean (SEM). To ensure objectivity, the outcome assessment and data analysis were performed by investigators who were blinded to the treatment groups using coded samples. All statistical analyses were performed using GraphPad Prism 9 (San Diego, CA, USA). One-way Analysis of Variance (ANOVA) was used to examine the significance between groups. When the significance level (*p*-value) was less than 0.5, Tukey’s Honestly Significant Difference (HSD) post hoc test was performed to identify specific group differences.

## Data Availability

The original contributions presented in this study are included in the article. Further inquiries can be directed to the corresponding authors.

## Abbreviations

The following abbreviations are used in this manuscript:

BPH	benign prostate hyperplasia
LUTS	lower urinary tract symptoms
pBOO	partial bladder outlet obstruction
QIE	*Quisqualis indica* extract
QA	quisqualic acid
TNFα	tumor necrosis factor-alpha
IL-6	interleukin 6
IL-1β	interleukin-1 beta
MCP-1	monocyte chemoattractant protein-1
TGF-β	transforming growth factor beta
α-SMA	alpha-smooth muscle actin
M_2_	muscarinic acetylcholine receptor M 2
M_3_	muscarinic acetylcholine receptor M 3
H&E	hematoxylin and eosin
OAB	overactive bladder
UAB	underactive bladder
